# Amino acid-dependent signaling *via* S6K1 and MYC is essential for regulation of rDNA transcription

**DOI:** 10.18632/oncotarget.10346

**Published:** 2016-06-30

**Authors:** Jian Kang, Eric P. Kusnadi, Allison J. Ogden, Rodney J. Hicks, Lukas Bammert, Ulrike Kutay, Sandy Hung, Elaine Sanij, Ross D. Hannan, Katherine M. Hannan, Richard B. Pearson

**Affiliations:** ^1^ Oncogenic Signaling and Growth Control Program, Peter MacCallum Cancer Centre, St Andrews Place, East Melbourne, Victoria, Australia; ^2^ Sir Peter MacCallum Department of Oncology, University of Melbourne, Parkville, Victoria, Australia; ^3^ Molecular Imaging and Targeted Therapeutics Laboratory, Cancer Therapeutics Program, Peter MacCallum Cancer Centre, East Melbourne, Victoria, Australia; ^4^ Institute of Biochemistry, Department of Biology, Swiss Federal Institute of Technology Zurich, Zurich, Switzerland; ^5^ Centre for Eye Research Australia, Royal Victorian Eye and Ear Hospital & Department of Ophthalmology, University of Melbourne, East Melbourne, Victoria, Australia; ^6^ Department of Biochemistry and Molecular Biology, University of Melbourne, Parkville, Victoria, Australia; ^7^ Department of Biochemistry and Molecular Biology, Monash University, Clayton, Victoria, Australia; ^8^ School of Biomedical Sciences, University of Queensland, Brisbane, Queensland, Australia; ^9^ Department of Cancer Biology and Therapeutics, The John Curtin School of Medical Research, The Australian National University, Canberaa, ACT, Australia

**Keywords:** amino acids, rDNA transcription, S6K1, MYC, Gerotarget

## Abstract

Dysregulation of RNA polymerase I (Pol I)-dependent ribosomal DNA (rDNA) transcription is a consistent feature of malignant transformation that can be targeted to treat cancer. Understanding how rDNA transcription is coupled to the availability of growth factors and nutrients will provide insight into how ribosome biogenesis is maintained in a tumour environment characterised by limiting nutrients. We demonstrate that modulation of rDNA transcription initiation, elongation and rRNA processing is an immediate, co-regulated response to altered amino acid abundance, dependent on both mTORC1 activation of S6K1 and MYC activity. Growth factors regulate rDNA transcription initiation while amino acids modulate growth factor-dependent rDNA transcription by primarily regulating S6K1-dependent rDNA transcription elongation and processing. Thus, we show for the first time amino acids regulate rRNA synthesis by a distinct, post-initiation mechanism, providing a novel model for integrated control of ribosome biogenesis that has implications for understanding how this process is dysregulated in cancer.

## INTRODUCTION

Mammalian cells rapidly and exquisitely regulate energy-consuming anabolic and energy-producing catabolic processes in response to altered levels of nutrients, growth factors, energy and oxygen in the environment. For proliferating cells, a major anabolic process is ribosome biogenesis, which is essential for protein synthesis, cell growth and proliferation. Given the high-energy demand to make new ribosomes, not surprisingly, ribosome biogenesis is tightly linked to cellular metabolism [1].

RNA polymerase I (Pol I)-dependent ribosomal RNA gene (rDNA) transcription is a key regulatory step in ribosome biogenesis. In mammalian cells, Pol I is responsible for transcribing the approximately 300 copies of the rDNA repeats [2], which constitutes 35-60% of all nuclear transcription [3]. rDNA transcription is initiated by the formation of a pre-initiation complex (PIC) at the promoter of active rDNA repeats in the nucleolus. The PIC consists of the upstream binding factor (UBTF, also called UBF), the TATA-binding protein (TBP)-containing complex selectivity factor 1 (SL-1) and Pol I [4]. An initiation-competent complex requires recruitment of RRN3 (also called TIF1A) whose activity is regulated by phosphorylation in response to various growth factors or stress stimuli [5, 6]. Pol I then dissociates from the promoter-bound initiation factors *via* promoter escape [7, 8]. Post-translational modifications of RRN3 are also required for conversion of the initiation-competent Pol I into the elongation competent form [9]. Pol I transcription generates the 47S pre-rRNA, which is rapidly processed to the 18S, 5.8S and 28S rRNAs in the nucleolus. These mature rRNAs, together with the 5S rRNA transcribed by Pol III, form the RNA backbone of the ribosome. rDNA transcription is commonly deregulated in cancer cells [10-12]. While there is no direct evidence that up-regulation of rDNA transcription is sufficient to drive malignant transformation, elevated rRNA synthetic activity, characterized by enlarged and/or increased numbers of nucleoli, is regarded as a feature of many cancers with potential prognostic value [13, 14]. Indeed, accelerated rDNA transcription is necessary for the survival of certain tumours and targeting Pol I transcription is proving to be a viable therapeutic approach for cancer treatment [15-19] with drugs inhibiting Pol I transcription now in phase I clinical trials. Given the critical link between cellular metabolism, ribosome biogenesis and cell growth, it is important to consider that as the solid tumor microenvironment is often poorly perfused due to inefficient neovascularisation, nutrient availability may be restricted [20]. Therefore, understanding how high rates of rDNA transcription and ribosome biogenesis are maintained in such a compromised environment will be important for identifying potential therapeutic targets for these cancers.

It is well established that growth factors acutely regulate Pol I transcription and, thus, ribosome biogenesis. This regulation is, in a large part, due to growth factor-dependent signaling *via* the phosphoinositide 3-kinase (PI3K)/AKT/mammalian target of rapamycin complex 1 (mTORC1) or RAS/RAF/ERK pathways. These pathways also form an intricate control network with the transcription factor, MYC, to impact on the functions of Pol I and its specific transcription factors [21-24].

Less is known about the regulation of ribosome biogenesis in response to amino acid abundance. One early report demonstrated that rRNA synthesis was down-regulated in response to amino acid starvation [25] and more recently James and Zomerdijk demonstrated that withdrawal of amino acids in HEK293 cells compromised the activation of Pol I transcription by insulin-like growth factor 1 (IGF-1) [26]. These studies did not elucidate the mechanism(s) by which amino acid availability regulated growth factor-dependent control of rRNA synthesis but implicated PI3K/mTORC1 activity being important for amino acids to stimulate rDNA transcription, independent of growth factors. Indeed, it is becoming apparent that mTORC1 can act as a hub linking the availability of amino acids to rDNA transcription [1]. Activated mTORC1 exerts its effect on Pol I transcription, at least in part, *via* the two key transcription factors RRN3 [6] and UBTF [27]. Upstream of mTORC1, accumulating evidence suggests that activation of mTORC1 by amino acids differs from that observed with growth factors. For example, it is independent on AKT and Tuberous sclerosis complex (TSC) [28], but requires Rag-GTPase or adenosine diphosphate ribosylation factor-1 (Arf1) GTPase as the key mediator [29-31]. Therefore, it is likely that growth factors and amino acids regulate rDNA transcription through distinct but overlapping mechanisms, possibly with mTORC1 acting as a critical hub that coordinates the cellular responses to both stimuli [1, 32, 33].

In the current study we undertook a detailed analysis of the mechanisms by which amino acid-regulation of rDNA transcription is mediated. Our findings thus provided novel fundamental insights into the nutrient control of ribosome biogenesis and cell growth *via* S6K1 and MYC that has implications for understanding how this process is deregulated in cancer cells. The results clearly implicate S6K1 as a potential key therapeutic target for treating cancers with limited nutrient availability and those driven by the oncogene MYC.

## RESULTS

### Amino acids regulate rRNA synthesis at multiple steps

**Figure 1:**
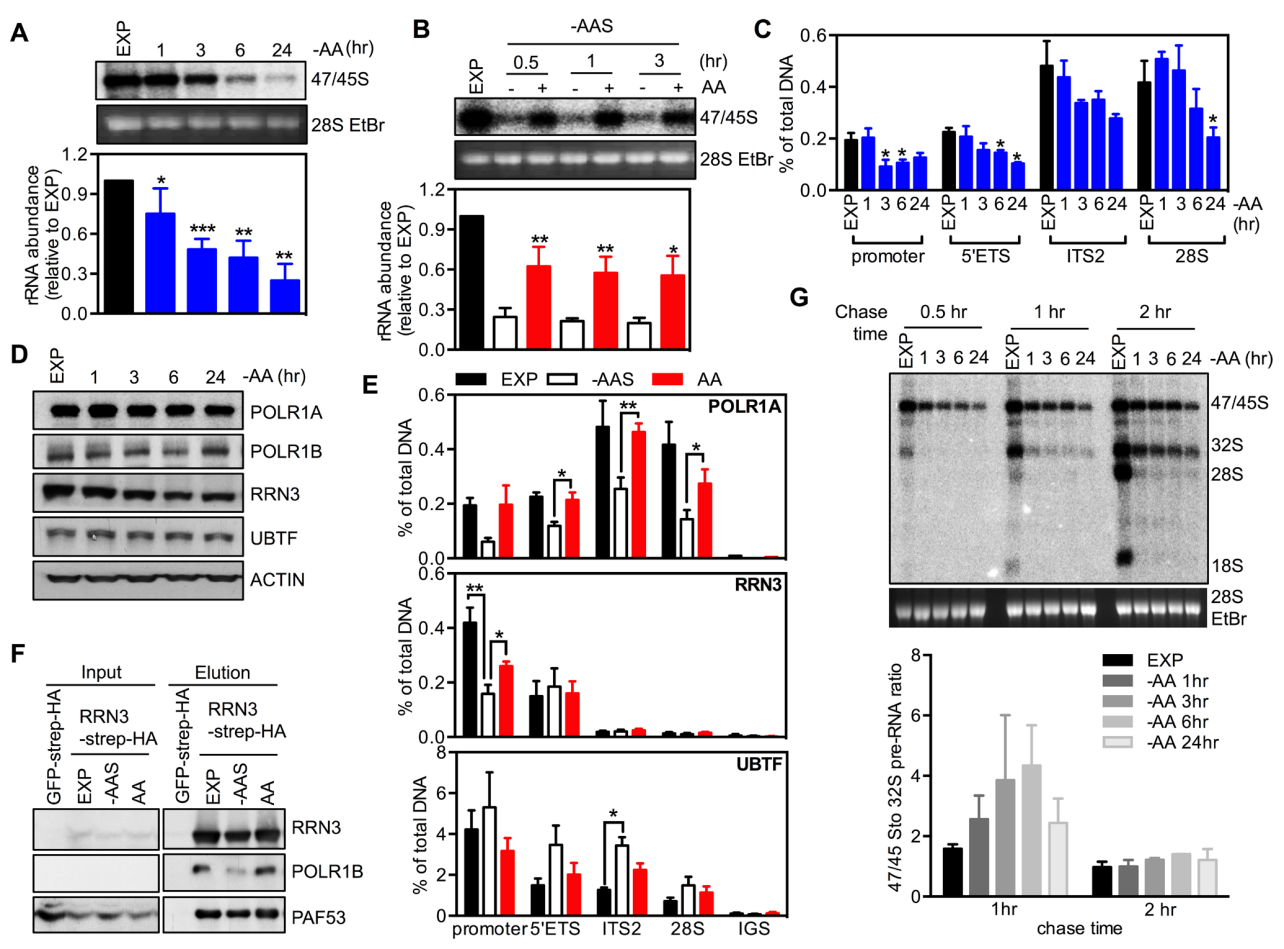
Amino acids regulate rRNA synthesis at multiple steps **A.** Exponentially growing HeLa cells (EXP) were starved of all amino acids (-AA) for times indicated. Cells were pulse labeled and 47S/45S rRNA synthesis analyzed. Representative images and resultant graph (mean +/− SEM) from *n* = 3-5 experiments. **B.** HeLa cells were starved in amino acid and serum starvation medium (-AAS) for 2 hours, then re-stimulated with all amino acids (AA) for the times indicated. Cells were pulse labeled and 47/45S rRNA synthesis analyzed. Representative images and resultant graph (mean +/− SEM) from *n* = 4 experiments. **C.**-**D.** HeLa cells were treated as in **A.** **C.** qChIP analysis assess Pol I loading on various regions of the rDNA. *n* = 3 experiments. * *p* < 0.05 compared to EXP cells. **D.** Immunoblotting with the indicated antibodies. Representative of *n* = 3 experiments. **E.** HeLa cells were starved of amino acid and serum (-AAS) for 2 hours, then re-stimulated with amino acids (AA) for 3 hours. qChIP analysis to assess POLR1A, RRN3 and UBTF loading on various regions of the rDNA. *n* = 3-4 experiments. **F.** HEK293 cells were treated as in **E.** Western blot analysis of immunoprecipitated ectopically expressed TAP-tagged RRN3 for endogenous POLR1B, and PAF53 in HEK293 cells. Representative of *n* = 3 experiments. **G.** HeLa cells were treated as in **A.** Cells were pulse labeled, chased for different time pointed as indicated and rRNA processing determined. Representative images and resultant graph (mean +/− SD) of *n* = 2 experiments. * *p* < 0.05, ** *p* < 0.01, ***p < 0.001 **A.** and **C.** compared to EXP cells **B.** compared to -AAS cells.

To determine the effects of altering amino acid abundance on rDNA transcription, a key regulatory step in ribosome biogenesis, we removed amino acids from the culture media of HeLa cells. The synthesis rate of 47/45S pre-rRNA was measured by ^32^P orthophosphate pulse labeling. Depletion of amino acids resulted in a rapid decrease in 47/45S pre-rRNA synthesis by approximately 25% (± 9%, *p* = 0.04) in one hour, 53% (± 3.4%, *p* = 0.00011) by 3 hours, which was reduced further by 6 and 24 hours (Figure 1A). To evaluate the ability of amino acids to modulate rDNA transcription independent of growth factors, HeLa cells were starved of amino acids and serum, then re-stimulated with amino acids alone. Under these conditions, re-addition of amino acids induced a significant increase in 47/45S pre-rRNA synthesis within 30 minutes (2.6 ± 0.1 fold, *p* = 0.008), which was maintained at both 1 and 3 hours after amino acid addition (Figure 1B). Similar effects of amino acid-induced 47/45S rRNA synthesis were observed in a second human cell line, the immortalized human BJ foreskin fibroblasts expressing h-TERT (BJ-T) (Figure S1A). Thus, our data indicates that in the absence of growth factors, re-addition of amino acids is sufficient to stimulate rDNA transcription.

We next measured Pol I binding across the rDNA to evaluate the effect of amino acid depletion on transcription initiation and elongation. Pol I binding was determined by quantitative chromatin immunoprecipitation (qChIP) using an antibody to POLR1A, the largest subunit of the Pol I complex. Pol I loading at the promoter and across the transcribed region of the rDNA (5′ETS, ITS2, 28S) did not change 1 hour after amino acid depletion (Figure 1C) even though a significant reduction in 47/45S rRNA synthesis was observed at this time point (Figure 1A). Thus the repression of Pol I transcription by acute (1 hour) amino acid withdrawal was not mediated exclusively by inhibition of pre-initiation complex formation, but more likely at subsequent steps such as promoter escape, elongation and/or global repression of all the steps simultaneously. However, by 3 hours of amino acid depletion, Pol I loading at the promoter region was significantly reduced suggesting Pol I transcription initiation was impaired at this time point (Figure 1C). Notably, no further repression in Pol I loading was observed at the longer time points (6-24 hours, Figure 1C) even though 47/45S rRNA synthesis continued to decrease with time (Figure 1A), suggesting that prolonged amino acid withdrawal impairs both Pol I transcription initiation and post-initiation processes (e.g., elongation). We further examined the protein abundance of key components of the Pol I transcription apparatus, specifically Pol I subunits (POLR1A, POLR1B), RRN3 and UBTF. Withdrawal of amino acids significantly reduced the expression of RRN3 after 6 hours, while the other protein expression levels remained unchanged (Figure 1D and Figure S1B). Since the interaction between RRN3 and Pol I/SL-1 is necessary for recruiting Pol I to the rDNA promoter [34], reduced RRN3 abundance suggests that suppression of rDNA transcription initiation by sustained amino acid withdrawal is associated with reduced abundance of the initiation-competent Pol I complex, at least at longer time points. However, as inhibition of rDNA transcription occurred within 1 hour of amino acid withdrawal, it is likely that these acute effects are predominantly due to altered activity of key components by post translational modification, as previously reported for growth factor stimulated rDNA transcription [5, 6, 27].

Consistent with amino acids being sufficient to modulate Pol I loading, re-addition of amino acids alone to cells that had sustained depletion of both amino acids and serum (-AAS) restored Pol I loading at the promoter and across the transcribed portion of the rDNA region (Figure 1E). Interestingly in this case there were no significant changes in the abundance of Pol I initiation complex components (Figure S1C). However, co-immunoprecipitation of overexpressed strep-HA tagged RRN3 demonstrated that the addition of amino acids increased the association between RRN3 and Pol I (POLR1B and PAF53, Figure 1F), and qChIP showed that RRN3 occupancy at the rDNA promoter was partially rescued by the addition of amino acids (Figure 1E), consistent with the post translational modifications of RRN3 that have been reported to modulate its interaction with Pol I/SL-1 [5, 6, 35, 36] being important for regulation of Pol I transcription initiation. Thus, in the absence of growth factors, re-addition of amino acids alone enhanced the interaction of RRN3 with Pol I, the assembly of Pol I initiation complex at the rDNA promoter and therefore transcription initiation. Compared to the restricted occupancy of RRN3 at the promoter and beginning of the transcript region, UBTF was found across all the tested regions of rDNA (Figure 1E). Interestingly, the association of UBTF with the rDNA 5′ETS and the transcribed region (Eg: ITS2) increased after amino acid and serum depletion while re-addition of amino acids displaced UBTF from the rDNA. UBTF binding to the rDNA has been reported to produce a nucleosome-like structure called the enhancesome, which prevents Pol I transcription elongation [37], whereas phosphorylation of UBTF by growth factor activating ERK signaling unfolds the enhancesome, allowing continued elongation [38, 39]. Our results provide support for the concept that amino acids mediating increasing pre-rRNA synthesis by enhancing transcription elongation *via* reducing UBTF binding to the rDNA.

To determine if modulation of amino acid abundance affects processing of the pre-rRNA into the mature 18S, 5.8S and 28S rRNAs, we performed a chase experiment with non-labeled medium after a 30 minute-pulse labeling of newly synthesized 47S pre-rRNA [40]. The intensity ratio of 47/45S to 32S pre-rRNA in amino acid-depleted cells increased after a 1 hour-chase compared to the exponentially growing cells, indicative of impaired early processing steps (Figure 1G). While this ratio returned to a level comparable to that of exponentially growing cells with a 2 hour-chase, the abundance of mature 28S and 18S rRNAs were barely detectable (Figure 1G), suggesting severe perturbation of the late processing steps. These results demonstrate that amino acid signaling is required for optimal processing of the 47S pre-rRNA. Consistent with amino acids controlling rRNA processing, removal of amino acids and serum virtually abolished 28S and 18S synthesis with re-addition of amino acids partially restored the stoichiometry between the processed rRNA species (Figure S1D). Taken together, our data suggests that amino acid withdrawal induces a rapid initial effect on Pol I transcription at both the post-initiation step and rRNA processing followed in the longer term by repression of Pol I recruitment to the rDNA promoter, most likely mediated by the reduction of the activity of Pol I initiation complex components.

Amino acids serve as important substrates for the tricarboxylic acid (TCA) cycle anaplerosis, which is critical for energy production and biosynthesis. Given the tight coupling of rDNA transcription to the cellular energy supply [35], a reduction of amino acid availability might indirectly modulate rDNA transcription through energy deficiency. However, compared to the rapid reduction of ATP abundance observed with glucose deprivation within3 hours, the ATP level was maintained for up to 6 hours after depletion of amino acids (Figure S1E), whereas by 1 hour rDNA transcription was significantly reduced (Figure 1A). These results suggest that the amino acid-mediated acute regulation of rDNA transcription and rRNA processing was not the result of cellular energy stress.

### Inhibition of rDNA transcription following amino acid depletion does not affect nucleolar integrity

Inhibition of rDNA transcription has been reported to induce visible changes in the nucleolar morphology and initiate a nucleolar stress response, which is associated with elevated activity of the tumour suppressor p53 [41]. Alterations in ribosome subunit production and cell growth can also induce distinct modes of nucleolar disruption [42, 43]. For example, inhibition of Pol I transcription leads to nucleolar segregation characterized by condensation and separation of nucleolar compartments including the nucleolar fibrillar center and granular component, and formation of “nucleolar cap” structures around the nucleolar remnant. On the other hand, alterations that affect the late processing steps of rRNA (such as 5-fluorouracil) have a minimal effect on nucleolar morphology [43].

**Figure 2:**
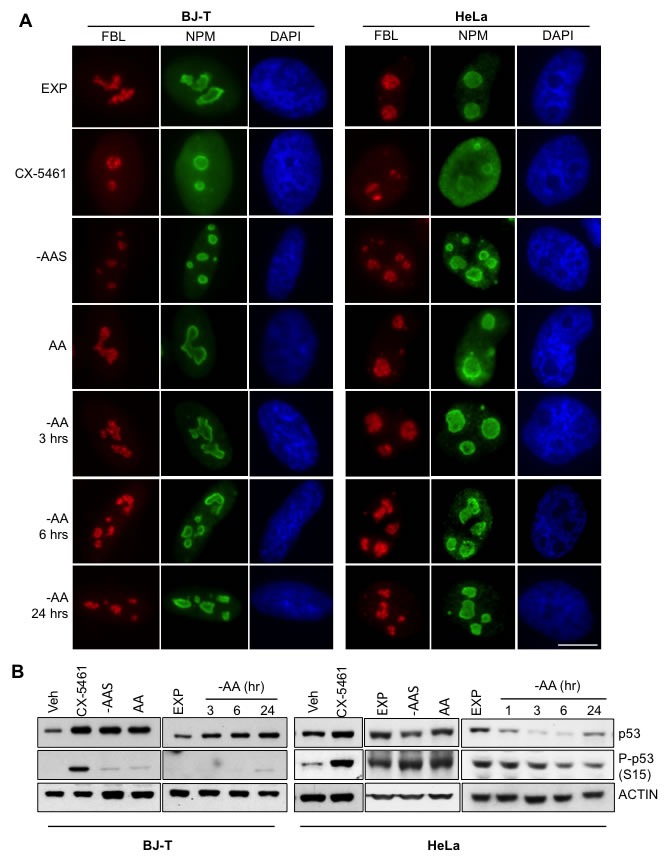
Inhibition of rDNA transcription following amino acid depletion does not affect nucleolar integrity **A.** BJ-T cells (left panel) or HeLa cells (right panel) were starved of all amino acids (-AA) for the times indicated or amino acid and serum starved (-AAS) for 2 hours and then re-stimulated with all amino acids (AA) for 3 hours. Alternatively exponentially growing (EXP) HeLa cells were treated with 1 μM CX-5461 for 1 hour. Representative images of immunostaining for fibrillarin (FBL: Red) and nucleophosmin (NPM: Green) with DAPI (Blue) counterstain of *n* = 2. Scale bar, 10 μm. **B.** BJ-T cells or HeLa cells were treated as in **A.** and protein lysates analyzed by immunoblotting for the indicated antibodies. Representative images from *n* = 2-4 experiments.

One hour treatment with CX-5461, a specific inhibitor of Pol I transcription [15, 16], caused nucleolar segregation characterized by fibrillarin (FBL) moving to “nucleolar caps” and the translocation of nucleophosmin (NPM) from the nucleolus into the nucleoplasm (Figure 2A). In contrast, the nucleolar integrity was maintained upon altering amino acid availability (Figure 2A) although the size of FBL positive foci was reduced in both the BJ-T and HeLa cells after sustained amino acid depletion (24 hours) and 5 hours of amino acid plus serum withdrawal (-AAS) (Figure S2A) while the foci number per nucleus tended to be increased (Figure S2B). p53 expression, phosphorylation and transcriptional activity were all increased in BJ-T cells starved for amino acids, consistent with the activation of a nucleolar stress response in the absence of nucleolar disruption (Figure 2B and Figure S2C). In contrast, HeLa cells, which have a defective p53 pathway due to binding of the human papillomavirus onconprotein E6 which increases its degradation [44], demonstrated reduced p53 expression and phosphorylation in response to amino acid withdrawal (Figure 2B). Thus, consistent with the role of amino acids in the late steps of rRNA processing (Figure 1G), amino acid depletion doesn't affect nucleolar integrity but induces p53 activation in p53-wild type cells.

### Regulation of rDNA transcription by amino acids is mediated through mTORC1 and its downstream target S6K1

**Figure 3:**
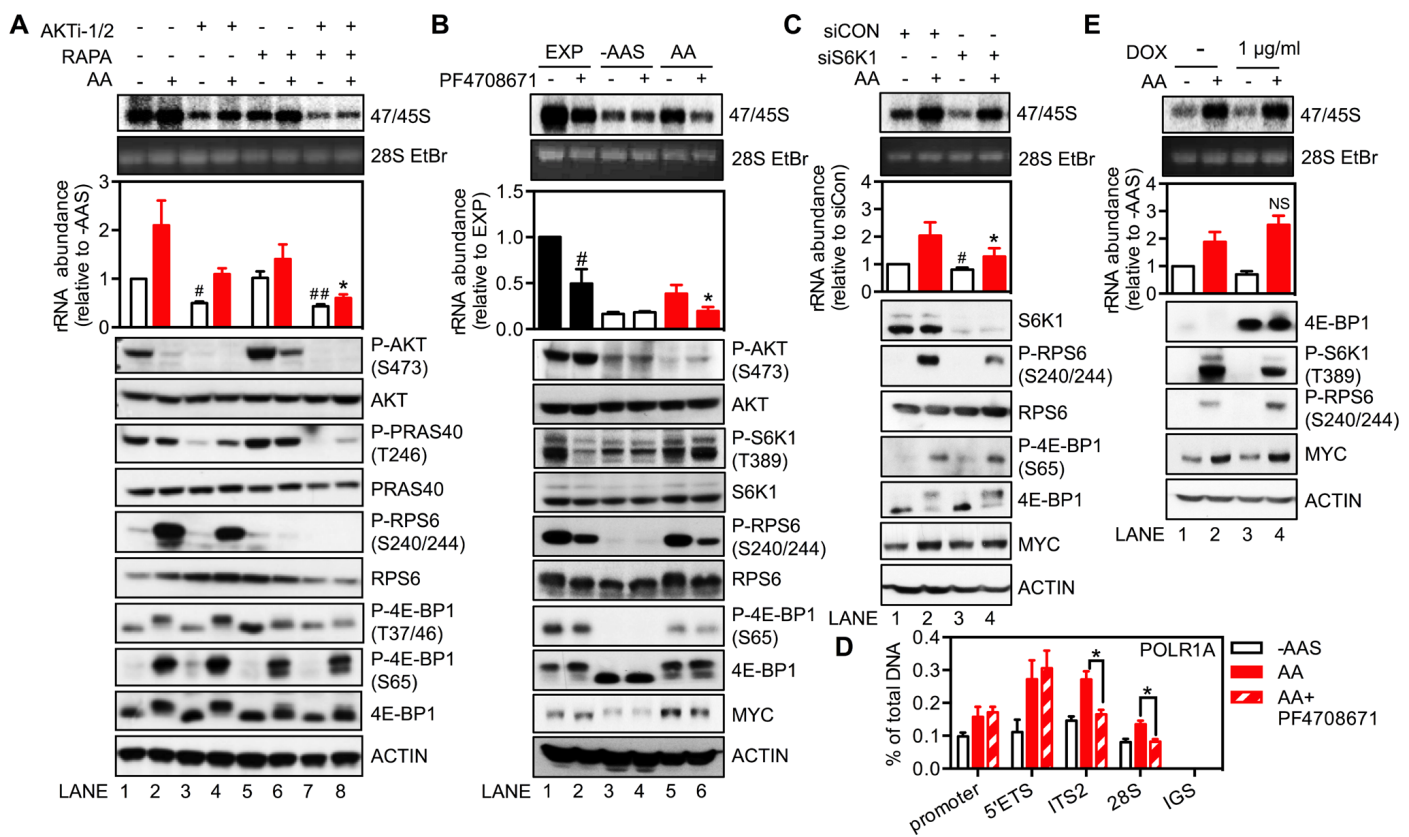
Regulation of rDNA transcription by amino acids is mediated through mTORC1 and the downstream target S6K1 **A.** and **B.** HeLa cells were amino acid and serum-starved (-AAS) for 2 hours, pre-treated with either 5 μM AKTi-1/2 or 20 nM rapamycin **A.** or 10 μM PF4708671 **B.** for 30 minutes, and then stimulated with all amino acids (AA) for 3 hours. **C.** HeLa cells were transfected with either non-targeting siRNA control (siCON) or pooled siRNA against S6K1 (siS6K1) for 3 days, and then starved of all amino acids and serum for 2 hours followed by amino acid stimulation for 3 hours. **D.** HeLa cells were treated as in **B.** **E.** HeLa cells stably expressing a doxycycline (DOX)-inducible 4E-BP1-4A mutant plasmid were treated with 1 μg/ml DOX for 24 hours, starved of all amino acids and serum for 2 hours and then stimulated with amino acids for 3 hours. **A.**-**C.** and **E.** In all cases cells were pulse labeled and 47/45S rRNA synthesis analyzed. Representative images and resultant graph (mean +/− SEM) of *n* = 3-4 experiments. Below the graph are representative immunoblotting images, *n* = 2-3. **D.** qChIP analysis to assess the Pol I loading on various regions of the rDNA. *n* = 3 experiments.**A.** *p < 0.05 compared to Lane 2; #*p* < 0.05, ##*p* < 0.01 compared to Lane 1. **B.** *p < 0.05 compared to Lane 5, ^#^*p* < 0.05 compared to Lane 1. **C.** *p < 0.05 compared to Lane 2, ^#^*p* < 0.05 compared to Lane 1. **D.** NS, not significant compared to Lane 2.

In order to evaluate the signaling pathways by which amino acids regulate rDNA transcription, the mTORC1 inhibitor rapamycin (RAPA) and AKT inhibitor (AKTi-1/2) [45] were utilized. Re-addition of amino acids to HeLa cells starved of amino acids and serum resulted in a two-fold increase in 47/45S pre-rRNA synthesis (Figure 3A, Lane 1 *vs*. Lane 2). Treatment of starved cells with AKTi-1/2 further reduced 47/45S rRNA synthesis by approximately 50% compared to untreated cells (Lane 1 *vs*. Lane 3) supporting a critical role for AKT activity in maintaining basal rDNA transcription rate. Re-addition of amino acids stimulated 47/45S pre-rRNA synthesis by approximately two-fold in the presence of AKTi-1/2 (Lane 3 *vs*. Lane 4), suggesting an AKT-independent component involved in the regulation of rDNA transcription by amino acids. None-the-less, the amino acid-stimulated rRNA synthesis was suppressed approximately 50% by inhibition of AKT (Lane 2 *vs*. Lane 4). In contrast, inhibition of mTORC1 by rapamycin had no significant effect on basal rDNA transcription rate in amino acid and serum-starved cells (Lane 1 *vs*. Lane 5) but did partially suppress the induction of 47/45S rRNA synthesis by amino acids (Lane 2 *vs*. Lane 6). Combining AKTi-1/2 and rapamycin (Lane 7 *vs*. Lane 8) resulted in additive suppression of both basal and amino acid-stimulated rDNA transcription. Similar trends were observed in the amino acid stimulated BJ-T cell system (Figure S3A). Taken together, this data shows that both AKT and mTORC1 are required for optimal amino acid-dependent rRNA synthesis, with AKT being required for maintaining basal rDNA transcription. This finding is consistent with the mTORC1-independent role of AKT in rDNA transcription identified in our previous study [40].

To investigate the distinct regulatory roles of AKT and mTORC1 further, we assessed the amino acid-dependent activity of each pathway. Re-addition of amino acids to starved HeLa cells failed to activate AKT, as assessed by phosphorylation of AKT and its substrate PRAS40 (Figure 3A bottom panel, Lanes 1 *vs*. Lane 2). In fact, AKT activity was reduced in response to amino acids, possibly mediated by the previously reported S6K1-IRS or S6K1-mTORC2 negative feedback loops [46-48]. AKTi-1/2 treatment further reduced basal AKT signaling, consistent with its inhibition of basal rRNA synthesis. Importantly, it did not affect mTORC1 re-activation in response to amino acids (Lane 3 *vs*. Lane 4), consistent with the AKT-independent-mTORC1 activation by amino acids. Unlike for AKT, re-addition of amino acids activated mTORC1 signaling, as evident by increased phosphorylation of ribosomal protein S6 (RPS6) and 4E-BP1. Inhibition of amino acid-induced mTORC1 activation by rapamycin was primarily associated with reduced S6K1 activity, with only a minor effect observed on the phosphorylation of 4E-BP1 (Lanes 5 *vs*. Lane 6). This is consistent with the previously reported preferential targeting of the S6K1 pathway by rapamycin [49, 50]. These results implicate mTORC1-dependent activation of S6K1 as a critical regulator of amino acid-mediated rDNA transcription.

To test this hypothesis, exponentially growing HeLa cells were treated with the S6K1 inhibitor PF4708671. Inhibition of S6K1 significantly reduced rDNA transcription in three hours (Figure 3B; Lane 1 *vs*. Lane 2). Importantly, PF4708671 suppressed the phosphorylation of RPS6 but did not alter the phosphorylation of AKT or 4E-BP1, suggesting a specific targeting of S6K1 signaling. As for rapamycin, PF4708671 treatment prevented amino acid-induced rDNA transcription (Lane 5 *vs*. Lane 6). Similar trends were also observed in the amino acid-stimulated BJ-T cell system (Figure S3B). Furthermore, siRNA mediated knockdown of S6K1 reduced phosphorylation of its substrate RPS6 without altering phosphorylation of 4E-BP1 (Figure 3C), and suppressed both basal rDNA transcription (Figure 3C, Lanes 1 *vs*. Lane 3) and 47/45S rRNA synthesis in the presence of amino acids (Lane 2 *vs*. Lane 4). Thus, both the inhibitor and knockdown approaches supported a role for S6K1 as a key molecule mediating amino acid-stimulated rDNA transcription. Moreover, inhibition of S6K1 signaling by PF4708671 did not reduce amino acids-dependent loading of Pol I at the 5′ETS of the rDNA (Figure 3D). In contrast, PF4708671 decreased loading of Pol I at the ITS2 and 28S regions of the gene, downstream of the 5′ETS, which suggested that inhibition of S6K1 signaling blocks amino acids-driven Pol I elongation but not transcription initiation, leading to accumulation of stalled Pol I at the 5′ end of the rDNA. Together, our results suggested S6K1 signaling is required for efficient rDNA transcription downstream of the 5′ETS region.

The other major downstream target of mTORC1 is 4E-BP1, which binds to and inhibits eIF4E, a rate-limiting initiation factor for cap-dependent translation. Phosphorylation of 4E-BP1 by mTORC1 leads to its dissociation from eIF4E thus allowing eIF4E to form an active initiation complex at the 5′ end of mRNAs. It has previously been reported that expression of the 4E-BP1-4A mutant mediates a dominant inhibitory effect on eIF4E activity, the result of which is to reduce translation of 5′-terminal oligopyrimidines (5′TOP) mRNAs [51]. Consistent with this finding, polysome profiling demonstrated that expression of the 4E-BP1-4A mutant, induced by doxycycline for 24 hours, inhibited translation of 5′TOP mRNAs as evidenced by *EEF1A1* and *RPS6* mRNAs shifting from the actively translating heavy polysomes to the lighter ones which have a reduced number of 80S ribosomes attached to the mRNA (Figure S3C). In contrast, expression of the 4E-BP1-4A mutant did not alter the ability of amino acids to stimulate 47/45S pre-rRNA synthesis (Figure 3D). Taken together with the lack of effect of PF4708671 or knockdown of S6K1 expression on the phosphorylation of 4E-BP1, our results clearly demonstrate that regulation of rDNA transcription mediated by amino acids is critically dependent on mTORC1 activated S6K1, but not on eIF4E-mediated translation.

### MYC is required for the response of rDNA transcription to amino acids

**Figure 4:**
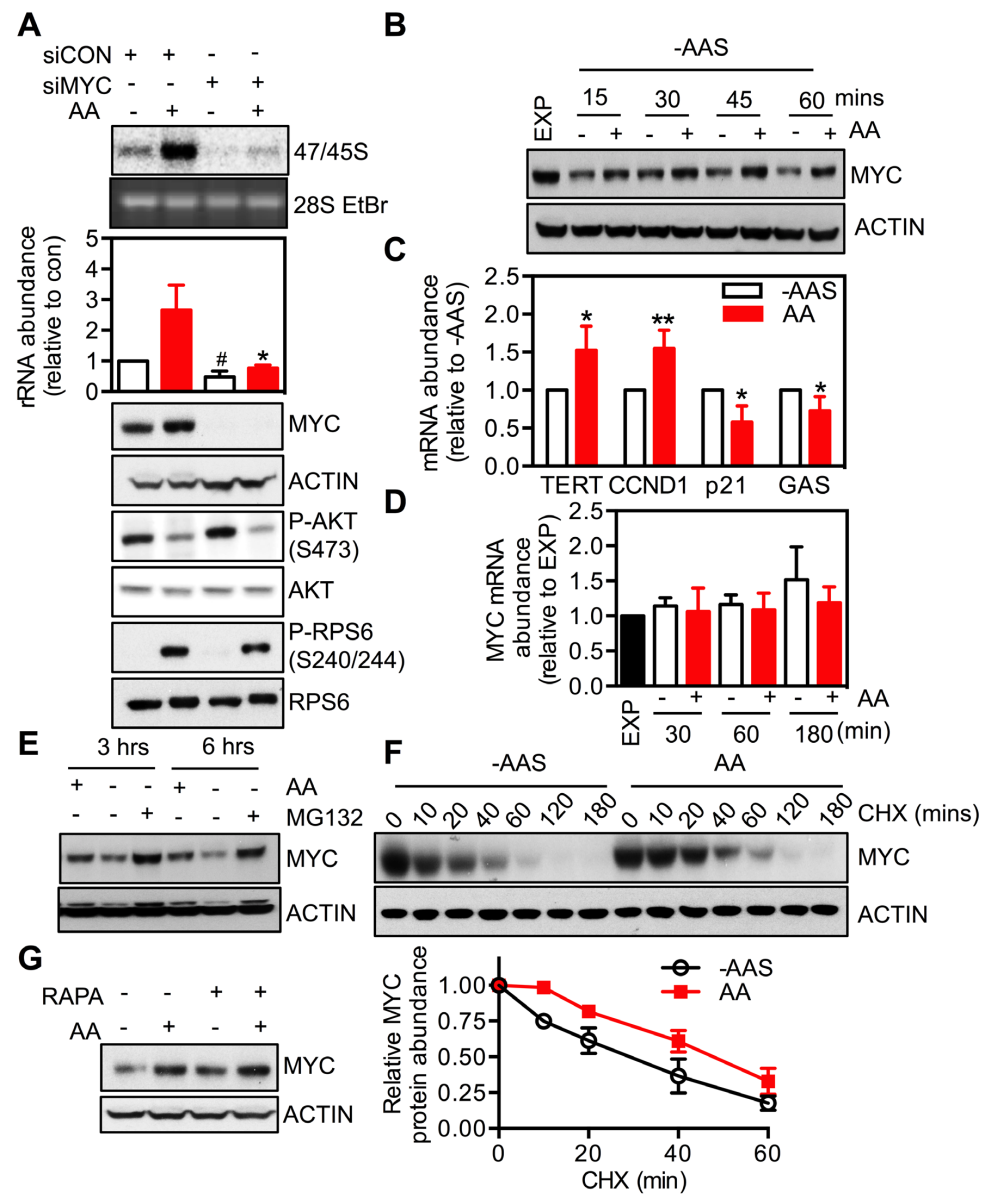
MYC is required for the response of rDNA transcription to amino acids **A.** HeLa cells were transfected with either non-targeting siRNA control (siCON) or pooled siRNA against MYC (siMYC) for 3 days, amino acid and serum-starved for 2 hours and then stimulated with amino acids (AA) for 3 hours. Cells were pulse labeled and 47/45S rRNA synthesis analyzed. Representative images and resultant graph (mean +/− SEM) of *n* = 3 experiments. Below the graph are representative immunoblotting images, *n* = 2. * *p* < 0.05 compared to Lane 2; # *p* < 0.05 compered to lane 4. **B.**-**D.** HeLa cells were amino acid and serum starved (-AAS) for 2 hours and then stimulated with all amino acids (AA) for the times indicated **B.** and **D.** or 3 hours **C.** Representative immunoblotting images **B.**, *n* = 2 blots) and quantitation of qRT-PCR for MYC and MYC transcriptional targets *TERT*, *CCND1*, *p21* and *GAS* **C.** and **D.**, *n* = 3-4 experiments). *p < 0.05, **p < 0.01 compared to -AAS cells.**E.** HeLa cells were deprived of amino acids in the presence of 10 μM MG132 for 3 or 6 hours. Representative immunoblotting images from *n* = 2 experiments. **F.** HeLa cells were amino acid and serum-starved for 2 hours and then stimulated with amino acids in the presence of 10 μg/ml cyclohexamide (CHX) for the times indicated. Representative immunoblotting images and quantitation (mean +/− SEM) from *n* = 3 experiments. **G.** HeLa cells were amino acid and serum-starved for 2 hours, pre-treated with 20 nM rapamycin for 30 minutes prior to 3-hour amino acid stimulation. Representative of *n* = 2 blots.

The oncogenic transcription factor MYC, is a well-characterized central regulator of ribosome biogenesis that not only interacts with the PI3K/AKT/mTORC1 pathway at multiple levels but also modulates growth factor-driven Pol I-dependent rDNA transcription [22, 24, 40]. However, whether MYC plays a role in amino acid-dependent regulation of rDNA transcription remains unknown. To address this question, we suppressed MYC expression in HeLa and BJ-T cells using siRNA. Depletion of MYC expression prevented amino acid-induced 47/45S rRNA synthesis both in the HeLa (Figure 4A) and BJ-T system (Figure S4A). Moreover the basal rDNA transcription rate in amino acid and serum-starved cells was also reduced by MYC knockdown (Figure 4A and Figure S4A) as expected due to a global role for MYC in the regulation of rDNA transcription [24].

The role of MYC in amino acid-driven rDNA transcription was further investigated by examining the effect of modulating amino acid availability on MYC expression and activity. Removal of amino acids and serum decreased MYC protein abundance, which was rapidly reversed by re-addition of amino acids in HeLa (Figure 4B) and BJ-T cells (Figure S4B). Moreover, re-addition of amino acids (AA) increased MYC transcriptional activity, as reflected by elevated (*TERT*, *CCND1*) or repressed (*p21*, *GAS*) expression of MYC target genes (Figure 4C). In contrast, MYC mRNA level did not change (Figure 4D), suggesting that the regulation of MYC expression by amino acids was at a post-transcriptional step.

Indeed the reduction of MYC protein abundance upon amino acid withdrawal was efficiently blocked by pre-treatment with the proteasome inhibitor MG132 (Figure 4E). Moreover re-addition of amino acids delayed the degradation of MYC protein even in the presence of the protein biosynthesis inhibitor cycloheximide (CHX) (Figure 4F). This suggests that, at least in part, up-regulation of MYC expression by amino acids is due to an increase in the protein's half-life. Taken together our results support the conclusion that modulation of MYC expression by amino acids may be an additional mechanism by which nutrient availability regulates rDNA transcription and ribosome biogenesis.

Studies from several groups, including our own, have demonstrated that mTORC1 signaling, *via* either 4E-BP1 or S6K1, promotes MYC translation [52-55]. We therefore investigated the interaction between mTORC1 and MYC signaling in amino acid-mediated control of rDNA transcription. Neither rapamycin (Figure 4G), inhibition of S6K1 (Figure 3B and 3C) nor expression of the dominant negative 4E-BP1 mutant (Figure 3E) altered MYC protein abundance in HeLa cells. Alternatively, knockdown of MYC did not alter mTORC1 activity as indicated by phosphorylation of RPS6 (Figure 4A and Figure S4A). These results suggest that mTORC1/S6K1 signaling and MYC transcriptional network, downstream of amino acids, target Pol I transcription through parallel pathways.

### Amino acid availability alters the response of rDNA transcription to growth factors

The coordination of signaling pathways initiated by nutrients and growth factors is essential for intracellular homeostasis and, consequently, optimal cell growth [1, 56]. A previous report of amino acid dependent stimulation of rDNA transcription by IGF-1 [26] led us to further evaluate the inter-reliance of these two signaling networks on the control of rDNA transcription. We demonstrated that amino acids stimulated rDNA transcription in the absence of serum (Figure 1B). We then examined the response of 47/45S pre-rRNA synthesis to growth factors (insulin:INS or dialyzed serum:DS) in the absence of amino acids to assess the requirement of amino acids for growth factor-induced Pol I transcription.

**Figure 5:**
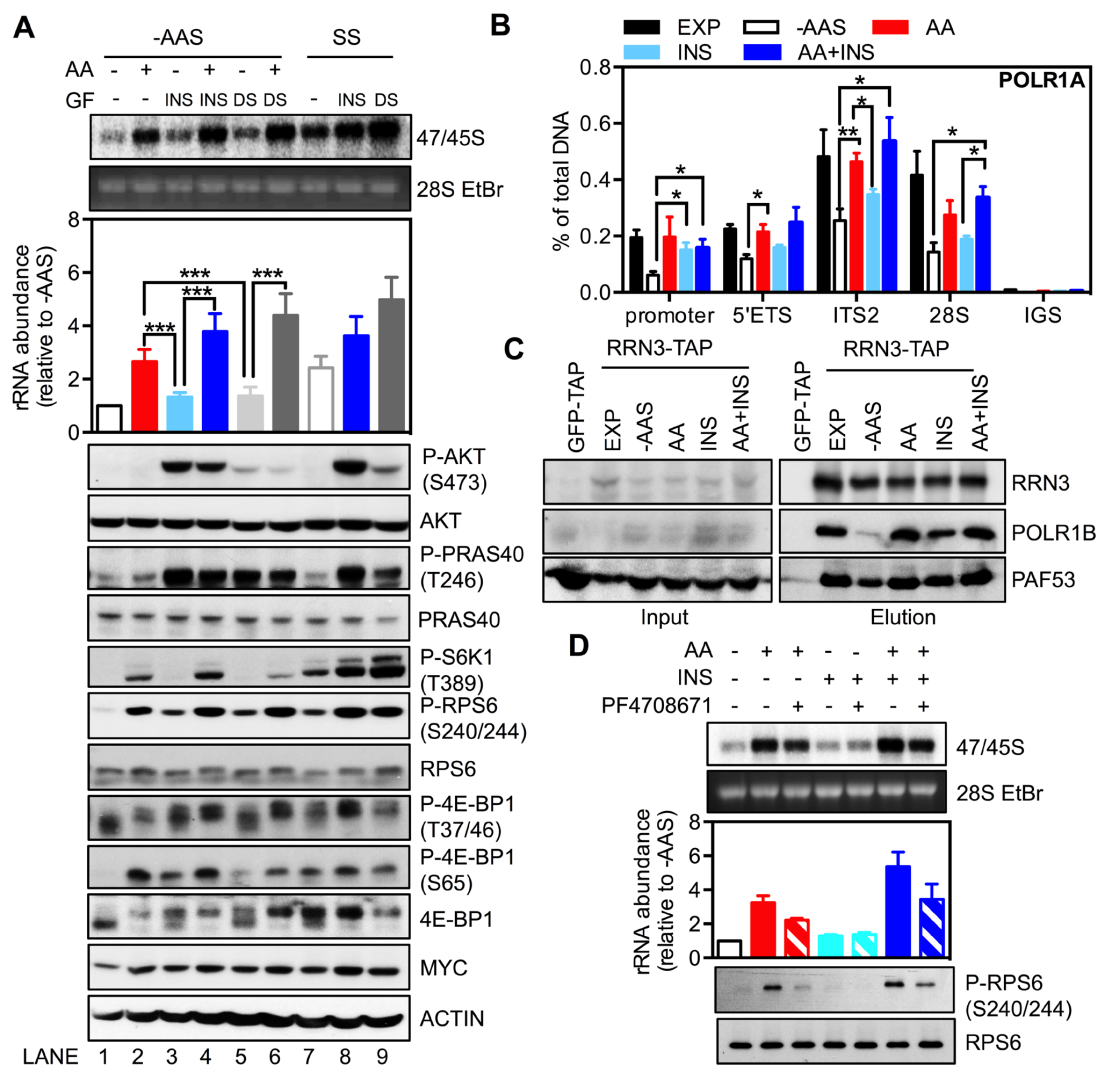
The availability of amino acids affects the response of rDNA transcription to growth factors **A.**-**B.** Exponentially growing HeLa cells (EXP) were starved of amino acids and serum (-AAS) for 2 hours and then stimulated with either amino acids (AA), 100 nM insulin (INS) or 10% dialyzed serum (DS), either alone or in combination as indicated, for 3 hours. Alternatively, HeLa cells were serum-starved (SS) for 24 hours then stimulated with 100 nM insulin or 10% dialyzed serum for 3 hours. **A.** Cells were pulse labeled and 47/45S rRNA synthesis analyzed. Representative images and resultant graph (mean +/− SEM) of *n* = 3 experiments. Below the graph are representative immunoblotting images, *n* = 2 experiments. **B.** qChIP analyses to assess Pol I loading on various regions of the rDNA. *n* = 3-5 experiments. **C.** HEK293 cells were treated as in **A.** Western blot analysis of immunoprecipitated ectopically expressed strep-HA-tagged RRN3 for endogenous POLR1B, and PAF53 in HEK293 cells. Representative images from *n* = 3 experiments.**D.** HeLa cells were amino acid and serum-starved for 2 hours, pre-treated with 10 μM PF4708671 for 30 minutes, and then stimulated with all amino acids for 3 hours. Cells were pulse labeled and 47/45S rRNA synthesis analyzed. Representative images and resultant graph (mean +/− SD) of *n* = 2 experiments. *p < 0.05, **p < 0.01, ***p < 0.001.

As expected, both insulin (Figure 5A top panel; Lane 8) and dialyzed serum (Lane 9) increased the synthesis of 47/45S pre-rRNA in serum-starved cells (Lane 7). In the absence of amino acids, growth factor-induced rDNA transcription was markedly impaired (Lane 3 *vs*. Lane 8; Lane 5 *vs*. Lane 9) and significantly lower than that achieved with amino acid re-addition in cells starved for amino acids and serum (Lane 3 or 5 *vs*. Lane 2). Furthermore, induction of 47/45S rRNA synthesis by growth factors was markedly enhanced in the presence of amino acids (Lane 3 *vs*. Lane 4; Lane 5 *vs*. Lane 6) and was comparable to that achieved in serum-starved cells (Lane 4 *vs*. Lane 8; Lane 6 *vs*. Lane 9). These results suggest that the availability of amino acids modulates growth factor-driven rDNA transcription, consistent with the data from HEK293 cells [26].

Despite an inability to stimulate rDNA transcription in the absence of amino acids, re-addition of insulin to cells starved of amino acids and serum did induce Pol I loading onto the rDNA promoter to a level similar to that observed in exponentially growing cells (Figure 5B). Consistently, the association of RRN3 and Pol I was partially enhanced by insulin and restored by amino acids or the combination (Figure 5C) without significant changes in the abundance of Pol I initiation complex components (Figure S5). Re-addition of amino acids and the combination with insulin also stimulated Pol I loading across the rDNA (Figure 5B), which correlated with an increased abundance of 47S rRNA (Figure 5A; Lane 2 and Lane 4). In comparison to the increase of Pol I occupancy on the promoter, insulin alone was incapable of rescuing Pol I binding across the transcribed region of the rDNA following starvation and was unable to efficiently stimulate 47S pre-rRNA synthesis (Figure 5A; Lane 3), consistent with amino acid signaling-dependent transcription elongation required for insulin-stimulated rRNA synthesis.

We further dissected the key signaling events downstream of amino acids and insulin. While the addition of insulin or dialyzed serum to amino acid and serum-starved cells had little effect on 47/45S pre-rRNA synthesis, phosphorylation of AKT and its substrate PRAS40 were increased to an extent comparable to those observed in the serum-starved cells (Figure 5A bottom panel; Lane 3 *vs*. Lane 8; Lane 5 *vs*. Lane 9). Furthermore, while the combination of insulin or dialyzed serum with amino acids markedly increased 47/45S rRNA synthesis, it did not alter the phosphorylation level of AKT (Lane 4 *vs*. Lane 3; Lane 6 *vs*. Lane 5). In contrast, activation of the mTORC1 substrates, S6K1 (phosphorylation of S6K1 and RPS6) or 4E-BP1 (phosphorylation at T37/46 and S65) by either insulin or dialyzed serum alone (Lane 3 and 5) was less pronounced compared to the combination treatment of amino acids and growth factors (Lane 4 and 6). This is consistent with other reports that amino acid deficiency renders mTORC1 refractory to growth factor stimulation [28, 57]. Importantly, unlike AKT, the activity of mTORC1/S6K1 signaling correlated with the abundance of 47/45S rRNA, reinforcing our findings that S6K1 is the critical component of the AKT/mTORC1 network required for amino acid-dependent rDNA transcription. Indeed, inhibition of S6K1 signaling by PF4708671 suppressed stimulation of rDNA transcription by amino acids alone and in combination with insulin (Figure 5D), suggesting that S6K1 signalling is required for cooperation between amino acids and growth factors to modulate rDNA transcription.

**Figure 6:**
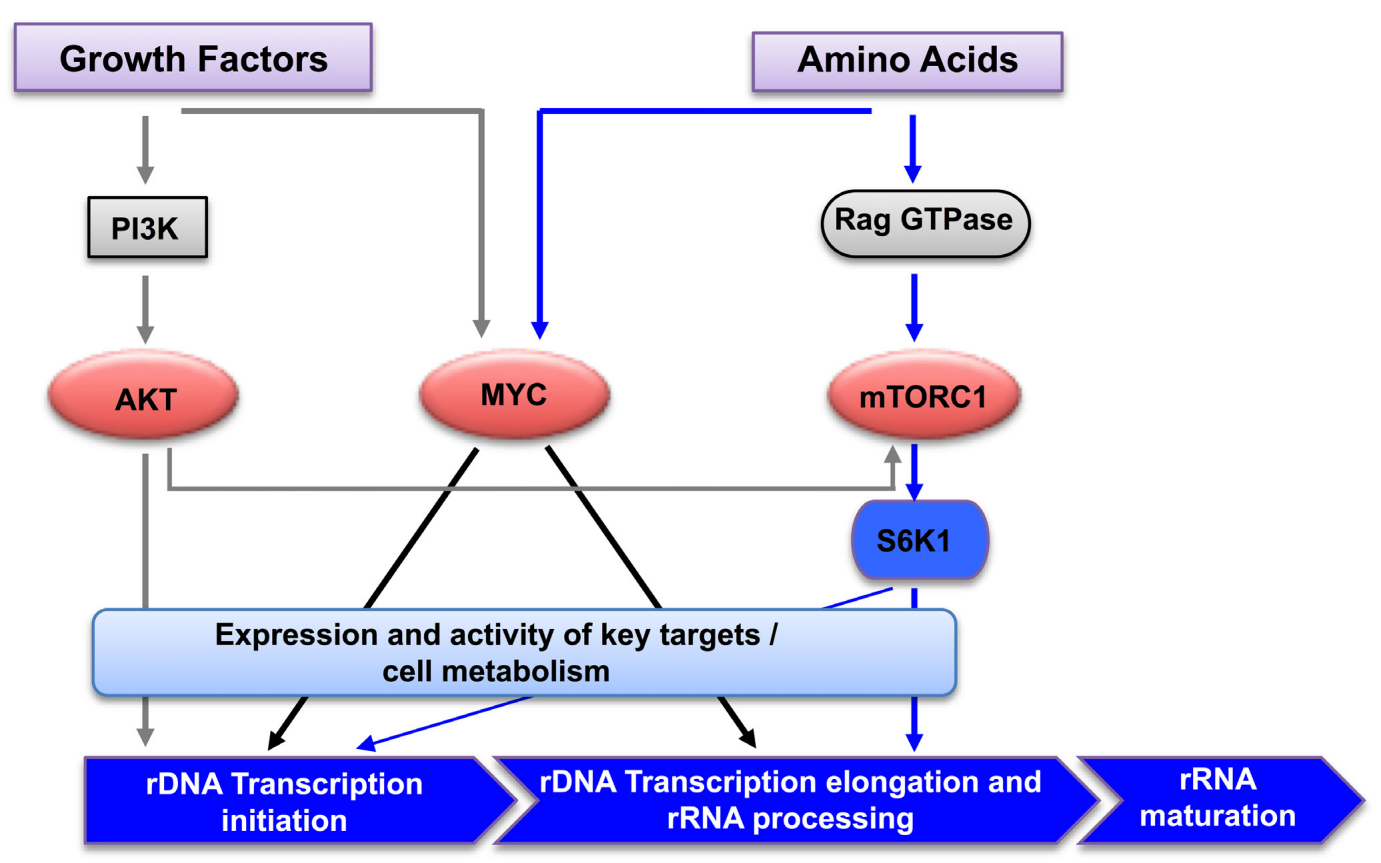
Regulation of rRNA synthesis by amino acids and growth factors Schematic diagram of the signaling network mediating cooperative regulation of rDNA transcription by amino acids and growth factors. Growth factor activation of AKT signaling primes rDNA transcription initiation, and amino acids promote rDNA transcription elongation, rRNA processing and the synthesis of mature rRNAs *via* mTORC1/S6K1 and MYC signaling.PI3K: Phosphotidylinositol 3- kinase; mTORC1: mammalian target of rapamycin complex 1; S6K1: ribosomal protein S6 kinase 1.

Our previous study demonstrated that AKT, independent of mTORC1, was required for growth factor-dependent initiation of rDNA transcription (Pol I loading onto the promoter), whereas mTORC1 was required for subsequent elongation and rRNA processing [40]. The data here further indicate that in the absence of amino acids, signaling downstream of growth factors (predominantly AKT signaling) stimulates initiation of rDNA transcription by increasing Pol I recruitment to the promoter, but the 47S pre-rRNA synthesis is significantly limited by impaired transcription elongation due to deficiency of mTORC1-dependent activation of S6K1. Efficient transcription of the rRNA gene thus relies on amino acid-driven mTORC1 signaling. Overall we propose a model in which growth factor-activated AKT signaling, in the absence of amino acids, is not sufficient to drive 47S pre-rRNA synthesis but does promote Pol I loading onto the rDNA promoter, i.e. initiation of transcription. On the other hand, amino acid-activated mTORC1 signaling is essential for post-initiation regulation of rDNA transcription at the stages of elongation and rRNA processing. Consequently cooperation between growth factor and amino acid signaling ensures optimal rRNA synthesis that also requires MYC activity (Figure 6).

## DISCUSSION

Amino acids serve as important anaplerotic substrates which are critical to support cell growth and proliferation by enabling energy production, sustaining macromolecule biosynthesis and maintaining redox homeostasis [58]. In a proliferating cell, the major energy consuming process is ribosome biogenesis, which is rate limiting for protein synthesis, cell growth and proliferation [59, 60]. Accordingly, it is likely that cells have evolved an intricate nutrient sensing network converging to modulate ribosome biogenesis.

The importance of in-depth analysis of this network is reinforced by the fact that metabolic reprogramming of cancer cells is one of the hallmarks of cancer. Tumour cells commonly become reliant on glycolysis, termed the “Warburg effect” and this is the basis of functional imaging of tumors using the glucose analogue, ^18^F-fluro-2-deoxyglucose (FDG) and positron emission tomography (PET). While most cancers have high FDG-avidity and there is generally a relationship between the intensity of FDG uptake and biological aggressiveness, some aggressive tumors, nevertheless, lack a significant increase in glycolytic flux. In such cancers, growth and proliferation must be fuelled by alternative substrates, with amino acids being the prime candidate, in particular glutamine [61]. Furthermore, many solid tumors thrive in a poorly vascularized microenvironment with limiting concentrations of growth factors and nutrients [20]. Thus we posit that elucidating how the metabolic network senses amino acid abundance and then controls ribosome biogenesis will provide novel targets for manipulating this process, which is emerging now as an important area for cancer treatment [62].

Here we demonstrate that S6K1 is the critical regulator of amino acid-driven rRNA synthesis. As a key downstream effector of mTORC1, S6K1 has been reported to cooperate with MYC to modulate rDNA transcription through RRN3 in Drosophila [63] and regulate the ribosome biogenesis transcriptional program, specifically by altering the transcription of nucleolar factors required for rRNA synthesis, cleavage, post-transcriptional modifications, ribosome assembly and transport [64]. Recently two groups independently demonstrated that mTORC1-S6K1 signaling promoted *de novo*-synthesis of pyrimidines and their incorporation into RNA and DNA, which is required for ribosome biogenesis and cell growth, in response to growth factors and nutrients [65, 66]. Thus, S6K1 is a key molecule linking both nutrient and growth factor signaling to rDNA transcription, suggesting that hyperactivation of S6K1 is a potential key driver of cancer cell growth in poorly perfused tumors with limiting nutrient availability.

The PI3K/AKT/mTORC1 pathway and the transcription factor, MYC, cooperate to regulate rRNA synthesis and ribosome biogenesis *via* the control of Pol I activity and expression of components of the Pol I complex and its specific transcription factors [21-24]. The data shown in Figures 3 and 4 demonstrate that manipulation of mTORC1 or S6K1 activity has no effect on MYC expression and conversely MYC expression does not regulate mTORC1/S6K1 signaling. These are important and novel results that are consistent with mTORC1/S6K1 signaling and the MYC transcriptional network targeting Pol I transcription through parallel pathways.

We recently defined a critical role of the mTORC1 upstream kinase AKT in the growth factor-mediated regulation of rDNA transcription initiation and a specific requirement for continuous mTORC1 activity for elongation [40]. Here we show that AKT-dependent, but mTORC1-independent, signaling is essential for maintaining the basal rDNA transcription rate in the absence of growth factors and nutrients. Consequently inhibition of AKT, without effects on mTORC1 activation, impairs the response of Pol I transcription to amino acids. The mechanism by which AKT regulates the basal rDNA transcription rate independent of mTORC1 remains unclear although numerous members of the Pol I complex contain AKT phosphorylation consensus sites that may be critical for their activity. Furthermore inhibition of AKT has been reported to be more potent than inhibition of mTORC1 in repressing amino acid-mediated 5′TOP mRNA translation [67]. This raises the possibility that AKT-driven mRNA translation could contribute to its regulation of Pol I transcription. Future analysis of the potential role of AKT in regulating the expression and activity of key molecules for Pol I transcription will be important for expanding our understanding of the control of ribosome biogenesis.

Coordinated regulation of multiple steps including rDNA transcription initiation and elongation, as well as rRNA processing is required for efficient synthesis of the rRNAs [68, 69]. By using qChIP analysis to evaluate Pol I and core Pol I transcription factors (RRN3 and UBTF) loading onto the rDNA in combination with pulse metabolic labeling to measure newly synthesized 47S pre-rRNA, we demonstrated that acute amino acid removal from exponentially growing cells inhibited 47S pre-rRNA synthesis and processing without changing Pol I loading onto the rDNA. This suggests that amino acid stimulation is required for the post-initiation steps and that the Pol I complexes are potentially stalled on the rDNA upon amino acid withdrawal. Consistent with this finding, in the absence of amino acids, growth factors were not sufficient to promote full length 47S pre-rRNA synthesis despite elevated Pol I loading at the rDNA promoter and enhanced RRN3-Pol I interaction, further supporting the essential role of amino acid signaling in regulation of post-initiation steps of rDNA transcription.

Together our results clearly demonstrate that signaling to rDNA transcription downstream of growth factors is distinct from that mediated by amino acids. Specifically, growth factors can optimally activate AKT, but not mTORC1, under amino acid-deficient conditions. Alternatively, amino acids alone potently activate mTORC1 independent of AKT. Collectively our data suggests that growth factor-activated AKT drives rRNA synthesis, primarily at the level of transcription initiation, whereas amino acid-activated mTORC1/S6K1 signaling primarily promotes transcription elongation and rRNA processing. It is likely that these distinct mechanisms of regulating rRNA synthesis in response to nutrients and growth factors might provide alternative control points that facilitate ribosome biogenesis in conditions favorable for cell growth and proliferation. Therefore the metabolic status of the cell is monitored by the checkpoints acting at both initiation and post initiation of rDNA transcription. Thus we hypothesize that rDNA transcription is primed by permissive signals from growth factor stimulation and before committing to the synthesis of full length of rRNA, cells sense the availability of nutrients to regulate the rate of transcription, primarily at the level of elongation and rRNA processing (Figure 6).

Furthermore, our data suggests that the uncontrolled growth of cancer cells under nutrient deficient conditions is, at least in part, underpinned by the ability of oncogenic signaling pathways such as AKT/mTORC1/S6K1 and/or oncogenes such as MYC, to maintain elevated ribosome biogenesis. The identification of the reliance on S6K1 for amino acid and growth factor-induced rDNA transcription implicates S6K1 as a potential key therapeutic target for treating cancers with limited nutrient availability that may be identified in the clinic, for example, through PET imaging. Given that animal studies have provided proof of principle that Pol I transcription can be a target for cancer therapy and Pol I transcription inhibitors are in phase 1 clinical trials, this study provides a compelling rationale for the design of more potent and specific drugs targeting ribosome biogenesis. Furthermore, the identification of distinct mechanisms of control of rDNA transcription raises the possibility of improving the potency of therapeutic approaches to target ribosome biogenesis by simultaneously targeting Pol I transcription initiation, elongation and rRNA processing.

## MATERIALS AND METHODS

### Cell lines, cell culture and reagents

Human HeLa cells were purchased from ATCC (Rockville, MD, USA) and cultured in Dulbecco's Modified Eagle Medium (DMEM) supplemented with 10% fetal bovine serum (Life technologies) and 2 mM L-glutamine at 37 °C in 5% CO_2_.

HeLa cells stably expressing the Tet-On inducible dominant-negative mutant of rat eIF4E-BP1 (pCW57.1-4E-BP1_4x alanine (A), a gift from David Sabatini [51], Addgene plasmid #38240), were generated by transfection with lipofectamine 2000 (Invitrogen) following the manufacturer's instructions, and selection with puromycin (1 μg/ml; Sigma) for two weeks. Doxycycline (Sigma) was used at 1gml for 24 hours to induce eIF4E-BP1-4A expression.

Cells were amino acid and serum starved by replacing medium with amino acid and serum starvation medium (1.4 mM CaCl_2_, 5.4 mM KCl, 0.8 mM MgSO_4_, 0.1 M NaCl, 1 mM NaH_2_PO_4_, 44 mM NaHCO_3_, 4.5 g/L glucose) supplemented with 4x Minimal Essential Medium (MEM) Vitamin solution (Life Technologies, #11120-052) for two hours. Cells were starved for amino acids by replacing growth medium with amino acid and serum starvation medium supplemented with 4x MEM Vitamin solution and 10% dialyzed fetal bovine serum (Life Technologies, #26400044). When indicated, cells were stimulated with all amino acids including 1x MEM amino acids, 1x MEM non-essential amino acids and 2mM glutamine (Life technologies, #11130-051, 11140-050 and 21051-024, respectively).

Inhibitors including: 5 μM AKTi-1/2 in dimethyl sulfoxide (DMSO) (MERCK, #124017), 20 nM rapamycin in ethanol (Calbiochem, #553210), 10 μM PF4708671 in DMSO (Sigma, #PZ0143), 40 μM 6-Diazo-5-oxo-L-norleucine (DON) in water (Sigma, #D2141). These were added 30 minutes prior to re-stimulation with amino acid solution. 10mM CX-5461 stocks (provided by Cylene Pharmaceuticals and Senhwa Biosciences, San Diego, CA, USA) were prepared in 50 mM NaH_2_PO_4_ (pH 4.5).

### siRNA transfection

Gene-specific siRNAs including *S6K1* (L-003616-00-0010), *MYC* (LQ-003282-02-0002), and ONTARGET Plus Non-Targeting siRNA control (siCON: D-001810-10-50) were purchased from Dharmacon (Millennium Science Pty Ltd., AUS). siRNA transfection was performed by reverse transfection, where cells were seeded directly onto plates containing transfection reagents and siRNA mixture as per the manufacturer's protocols.

### RNA extraction and RT-PCR

RNA was extracted either using the ISOLATE II RNA mini Kit (Bioline) or RNeasy Mini Kit (Qiagen) following the manufacturer's instructions. RNA concentration was quantitated using the NanoDrop ND1000 Spectrophotometer (Biolab).

First-strand complementary DNA (cDNA) was synthesized using random hexamer primers (Promega) and Superscript III (Invitrogen) per the manufacturer's instructions. qRT-PCR was performed in triplicate using FAST SYBR Green dye and primers (Supplementary Table S1) on the ABI StepOnePlus (Applied Biosystems). Data was analyzed using the ΔΔCT method.

### Western blotting and immunofluorescence

Protein lysates were harvested as described previously [40]. 10 −30 μg of lysate was separated by SDS-polyacrylamide gel electrophoresis (SDS-PAGE) prior to transfer to polyvinyl difluoride membranes. The membranes were incubated with the following primary antibodies obtained from Cell Signaling: AKT (# 9272), P-AKT (S473, # 4058), PRAS40 (# 2610), P-PRAS40 (T246, # 2997), P-p53 (S15, #9284), RPS6 (# 2217), and P-RPS6 (S240/244, # 2215), S6K1 (# 9202), P-S6K1 (T389, # 9205), 4E-BP1 (# 9452), P-4E-BP1 (T37/46, # 2855) and P-4E-BP1 (S65, # 9456). ACTIN (# 691002) was obtained from MP Biomedical. MYC (# ab32072) was obtained from Abcam. RRN3 antibody was provided by Prof Brian McStay, National University of Ireland, Galway, Ireland. POLR1B antibody was provided by Prof Lawrence Rothblum, University of Oklahoma Health Sciences Center, Oklahoma City, USA. p53 (sc-126) and p21 (sc-397) were obtained from Santa Cruz Biotechnology. The secondary antibodies were horseradish peroxidase-conjugated goat anti-rabbit (BioRad, # 1706515) or anti-mouse antibodies (BioRad, #1706516). Specific proteins were visualized by chemiluminescence with Western Lighting Plus ECL kit (Perkin-Elmer, Rowville, VIC, Australia). Densitometry was performed using the software Image.

Tandem affinity purification (TAP) was performed as described previously [70]. In brief, HEK293 cells overexpressing TAP-tagged RRN3 were cultured in DMEM supplemented with 10% FBS and 2 mM L-glutamine at 37 °C in 5% CO_2_. Cells were harvest after Tetracycline (Calbiochem) induction at 25 ng/ml for 48 hours. TAP-tagged RRN3 was immunopurified using Strep-Tactin Sepharose (IBA, 2-1201-010), eluted with 2.5mM d-Desthiobiotin (Sigma, D1411), and recaptured on monoclonal anti-HA-Agarose (Sigma, A2095). Elutes were subjected to immunoblotting.

For immunofluorescence, cells were fixed in 4% paraformaldehyde at room temperature for 10 minutes, washed with PBS, and blocked with 5% BSA in PBS and 0.3% Triton X-100 for 1 hour at room temperature. Cells were sequentially incubated with rabbit anti-Fibrillarin polyclonal antibody (Abcam, # ab5821) and Alexa Fluor 594 goat anti-rabbit secondary antibody (Life technologies, #A-11012). Stained cells were counterstained with prolong Gold antifade reagent with DAPI (Life Technologies, # P36935). Images were acquired on an Olympus BX-61 microscope equipped with a SPOT RT camera and the SPOT Advanced software (SPOT imaging Solutions, MI, USA).

### Pulse labeling and analysis

Pulse labeling to determine rRNA abundance was performed as described by Stefanovsky *et al*. [71]. Briefly, cells were labelled with 0.5 mCi ^32^P orthophosphate (PerkinElmer, NEX053C025MC) for 30 minutes prior to either harvest or “chase”. Chase experiments required a further 3 hours of incubation in label free medium (including inhibitors) before harvest. Equal RNA (2 to 5 μg) was separated on a 1.2% MOPS formaldehyde gel, which was visualized using ethidium bromide and the Gene Genius Bioimaging System (Syngene). The gel was dried (Model 583 Gel Drier) and exposed to a phosphorimager screen overnight. Bands corresponding to rRNAs were visualized with the Storm 820 Phosphorimager and intensities quantitated with ImageQuant software (GE Healthcare).

### Quantitative chromatin immunoprecipitation (qChIP)

qChIP was performed as described previously [40]. Briefly 6-7×10^6^ cells were cross-linked with 0.6% formaldehyde, and assays were performed with 15 μl of pre-immune rabbit sera or rabbit anti-POLR1A polyclonal antibody (obtained from Professor Larry Rothblum, University of Oklahoma Health Sciences Centre, Oklahoma City, USA). Samples were analyzed in triplicate using the FAST SYBR Green dye and primers (Supplementary Table S1) on the ABI StepOnePlus (Applied Biosystems). To calculate the percentage of total DNA bound, unprecipitated input samples from each condition were used as a reference for all qRT-PCR reactions.

### Statistics

Data and statistical significance was assessed using Student's t test in the GraphPad Prism software (Version 6, La Jolla, CA, USA). In experiments with *n* > 3, the graphs represent mean ± standard error of the mean (SEM) and when *n* < 3, the graphs represent mean ± standard deviation (SD). *p* values < 0.05 were deemed significant (*p* < 0.05: *; *p* < 0.01: **; *p* < 0.001: ***). *P* values > 0.05 were deemed not significant (ns).

## SUPPLEMENTARY MATERIALS FIGURES AND TABLE


