# Supplementary material for: Amino acid-dependent signaling via S6K1 and MYC is essential for regulation of rDNA transcription
Source: Oncotarget. 2016 Jun 30;7(31):48887–904. doi: 10.18632/oncotarget.10346 (PMC5226478; doi:10.18632/oncotarget.10346)
Supplement: Supplementary file 1 [file oncotarget-07-48887-s001.pdf]

Amino acid-dependent signaling *via* S6K1 and MYC is essential for regulation of rDNA transcription

Supplementary Material

MATERIALS AND METHODS

Cell lines and cell culture

Human BJ foreskin fibroblast cells immortalized by the expression of human telomerase reverse transcriptase (hTERT), the catalytic subunit of telomerase (BJ-T) were kindly supplied from Robert Weinberg *(68)*. BJ-T cells were cultured in a 4:1 ratio (v/v) mix of DMEM and Medium 199 supplemented with 15% fetal bovine serum (FBS) and 2 mM L-glutamine at 37°C in 5% CO_2. Cells were deprived of serum for 24 hours and then amino acid starved by replacing the medium with Krebs-Ringer Bicarbonate Buffer (KRBB; 25 mM HEPES, 111 mM NaCl, 25 mM $NaHCO_3$, 5 mM KCl, 2.5 mM $CaCl_2$ and 1 mM $MgCl_2$) supplemented with glucose (4.5 g/L) and insulin (100 nM) for 2 hours. When indicated, cells were stimulated with all amino acids including 1x MEM amino acids plus 1x MEM non-essential amino acids and 2mM glutamine (Life technologies).

Measurement of intracellular ATP levels

ATP was measured in the cell lysates using a colorimetric assay according to the manufacturer's instructions (Abcam, #ab83355). The data was normalized to the total protein content of the cell lysate as determined by DC protein assay (BioRAD) using bovine serum albumin as the standard.

Fractionation and quantitation of ribosomal profiles

HeLa cells stably expressing a doxycycline–inducible 4E-BP1-4A mutant plasmid were treated with 1 µg/ml doxycyline (DOX) for 24 hours, then with 50 µg/ml cyclohexamide for 15 minutes before harvested in hypotonic lysis buffer (1.5 mM KCl, 2.5 mM $MgCl_2$, 5 mM Tris pH 7.4, 50 µg/ml cyclohexamide, 0.5% Triton X-100, 0.5% deoxycholate, 3 mM DTT, RNAsin 40 U/ml, 1x complete protease inhibitor (Roche Applied Science, IN)). Fractionation and quantification of the ribosome subunits was performed as described in (4). Briefly, lysates from equal cell number (~1 mg) were loaded on 14 ml continuous 10-40% (w/v) sucrose gradients generated using an ISCO gradient maker. Samples were separated by centrifugation (SW41, 36000 rpm, 2 hours), fractionated (1ml fractions) using the Foxy Jr fraction collector and the absorbance at 260 nm determined with the ISCO UA-6 Absorbance detector (Teledyne, Thousand Oaks, CA, USA). RNA was extracted using RNeasy Mini Kit (Qiagen) following the manufacturer's instructions. cDNA synthesis and qRT-PCR were performed as described in the "Material and Methods" section. The primers are listed in Supplementary Table 1.

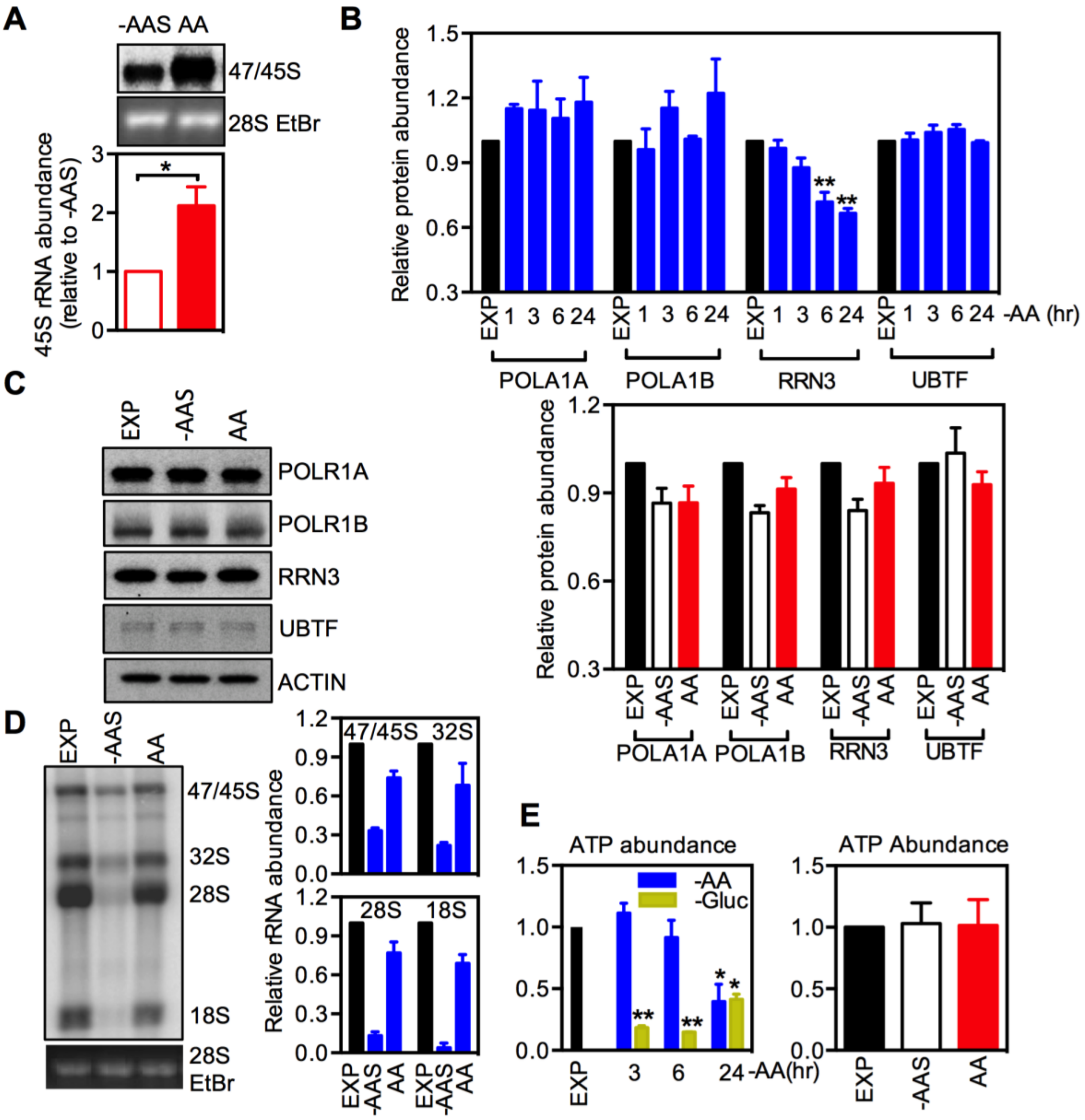

Supplementary Figure S1: Amino acids regulate rRNA synthesis at multiple steps. A. BJ-T cells were deprived of serum for 24 hours followed by amino acid starvation for 2 hours (-AAS). Cells were then stimulated with all amino acids (AA) for 3 hours. Cells were pulse labeled and 47/45S rRNA synthesis analyzed. Representative images and resultant graph (mean +/- SEM) for $n = 3$ experiments. $*p < 0.05$. **B.** Exponentially growing HeLa cells (EXP) were starved of all amino acids (-AA) for the times indicated. Representative images of immunoblotting are shown in Figure 1D and the graph represents mean +/- SEM for $n = 3$ experiments. $**p < 0.01$ compared to EXP. **C.** and **D.** HeLa cells were starved in amino acid and serum starvation medium (-AAS) for 2 hours and then re-stimulated with amino acids (AA) for 3 hours. (C) Immunoblotting with the indicated antibodies. $n = 3$ experiments. (D) Cells were pulse labeled, chased and rRNA processing determined. Representative images and resultant graph (mean +/- SEM) for n=3 experiments. (E) HeLa cells were either deprived of all amino acids (-AA) for the times indicated (left panel), or treated as in (C) (right panel). Samples were analyzed for ATP abundance. Graph represents mean +/-SEM for n=3 experiments. * $p < 0.05$; ** $p < 0.01$ compared to EXP.

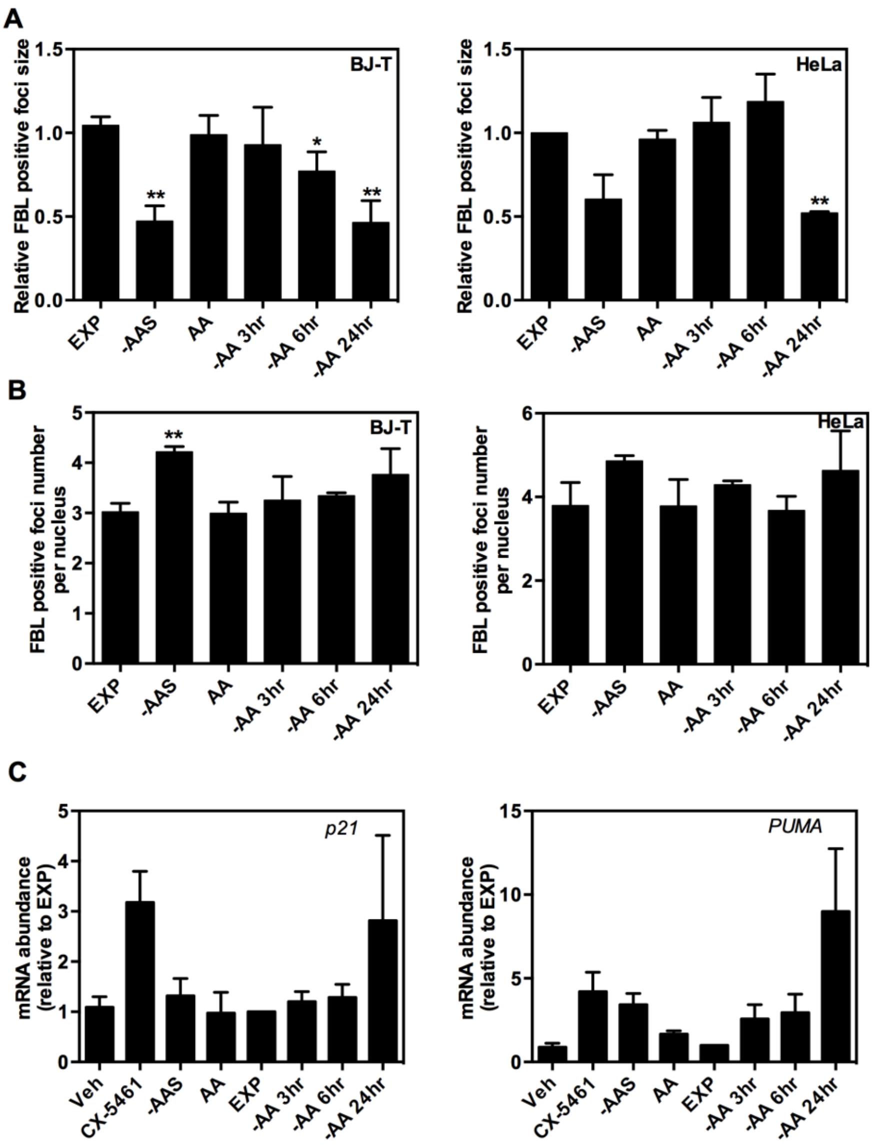

Supplementary Figure S2: Inhibition of rDNA transcription following amino acid depletion does not affect nucleolar integrity. A.- B. BJ-T (left panel) and HeLa (right panel) cells were starved of all amino acids (-AA) for the times indicated or amino acid and serum starved (-AAS) for 2 hours, then re-stimulated with all amino acids (AA) for 3 hours. Alternatively exponentially growing (EXP) HeLa cells were treated with 1 μM CX-5461 for 1 hour. Representative images of immunostaining for fibrillarin, nucleophosmin and DAPI are shown in Figure 2. Quantitation of the size of fibrillarin positive foci (A) or the number of fibrillarin positive foci per nucleus. **C.** qPCR analysis of *p21* and *PUMA* mRNA abundance in BJ-T cells. Graph of mean +/- SEM for *n* = 3 experiments.

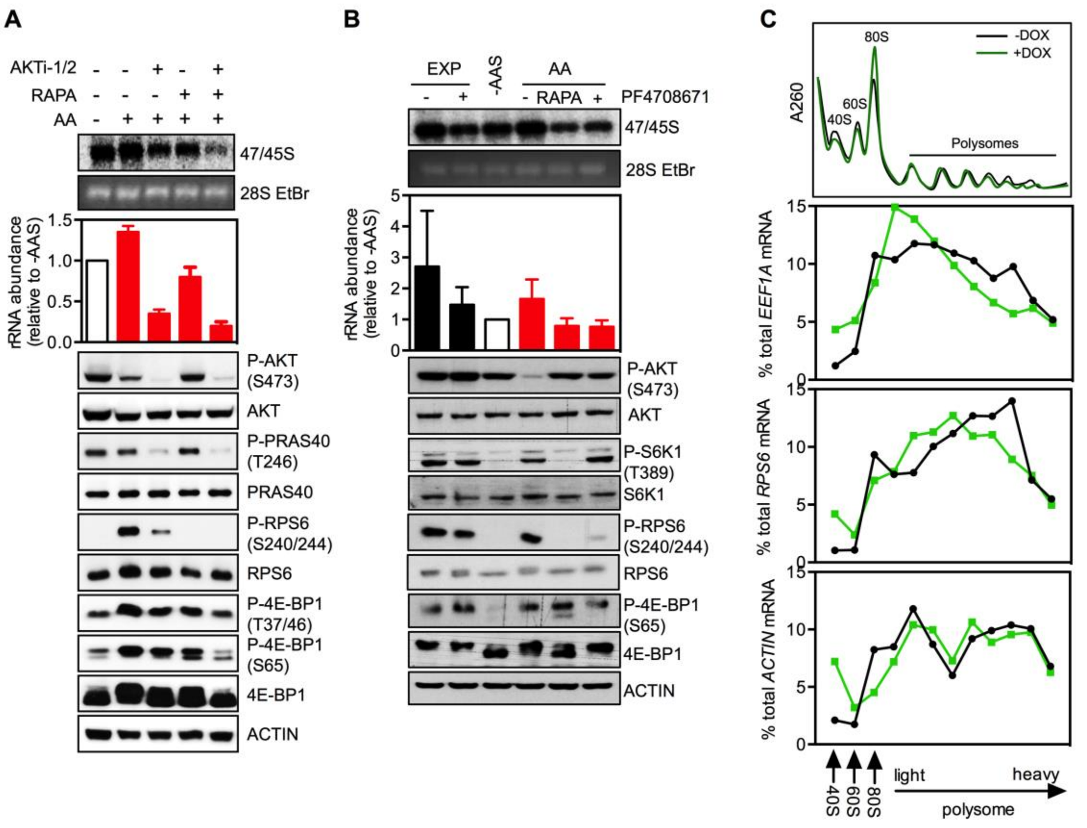

Supplementary Figure S3: Regulation of rDNA transcription by amino acids is mediated through mTORC1 and the downstream target S6K1. A.- B. BJ-T cells were deprived of serum for 24 hours followed by amino acid starvation for 2 hours. Cells were pre-treated with either 5 μM AKTi-1/2 or 20 nM rapamycin (A), or 10 μM PF4708671 (B) for 30 minutes and then amino acid stimulated for 3 hours. Cells were pulse labeled and 47/45S rRNA synthesis analyzed. Representative images and resultant graph (mean +/- SEM) for *n* = 3-4 experiments. Below the graph are representative immunoblotting images for the indicated proteins from *n* = 3 experiments. **C.** HeLa cells stably expressing the doxycycline (DOX)-inducible 4E-BP1-4A mutant were treated with 1 μg/ml DOX for 24 hours. Lysates from equal cell number (about 1mg) were separated on 10-40% sucrose gradients. Fractions were analyzed by qRT-PCR for the indicated 5’TOP (*EEF1A1* and *RPS6*) and non-5’TOP (*ACTIN*) mRNAs. Upper panel is a representative trace and lower panels are representative qRT-PCR analysis, *n* = 2.

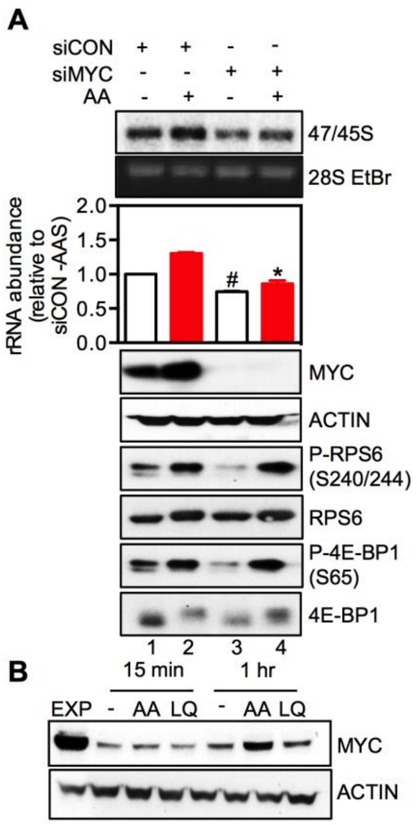

Supplementary Figure S4: MYC is required for the response of rDNA transcription to amino acids. A. BJ-T cells were transfected with either non-targeting siRNA control (siCON) or pooled siRNA against MYC (siMYC) for 3 days. Cells were then deprived of serum for 24 hours followed by amino acid starvation for 2 hours before re-addition of amino acids (AA) for 3 hours. Cells were pulse labeled and 47/45S rRNA synthesis analyzed. Representative images and resultant graph (mean +/- SEM) for $n = 3$ experiments. Below the graph are representative immunoblotting images for the indicated proteins from $n = 2$ experiments. *$p<0.05$ compared to Lane 2, $^{\#}p < 0.05$ compared to Lane 1. **B.** BJ-T cells were deprived of serum for 24 hours followed by amino acid starvation for 2 hours. Cells were then stimulated with amino acids (AA) or leucine plus glutamine (LQ) for 15 minutes or 1 hour. Representative immunoblotting images for $n = 2$ experiments.

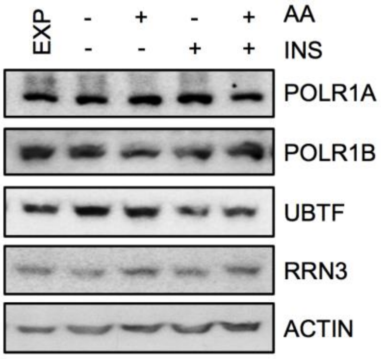

Supplementary Figure S5: The availability of amino acids affects the response of rDNA transcription to growth factors. Exponentially growing HeLa cells (EXP) were starved of amino acids and serum for 2 hours and then stimulated with either amino acids (AA) or 100 nM insulin (INS), either alone or in combination as indicated, for 3 hours. Protein lysates were analyzed by immunoblotting with the indicated antibodies. Representative immunoblotting images for *n* = 2 experiments.

Table S1: RT-PCR primers

Human rDNA promoter	Forward	CCCGGGGGAGGTATATCT TT
	Reverse	CCAACCTCTCCGACGACA
Human 5’ETS	Forward	GGCGGTTTGAGTGAGACGAGA
	Reverse	ACGTGCGCTCACCGAGAGCAG
Human ITS2	Forward	GAGAGAGACGGGGAGGGCGG
	Reverse	CCG AGG GAG GAA CCC GGA CC
Human 28S	Forward	AGTCGGGTTGCTTGGGAATGC
	Reverse	CCCTTACGGTACTTGTTGACT
Human MYC	Forward	CAGCTGCTTAGACGCTGGATT
	Reverse	GTAGAAATACGGCTGCACCGA
Human TERT	Forward	TGACACCTCACCTCACCCAC
	Reverse	CACTGTCTTCCGCAAGTTCAC
Human CCND1	Forward	CCAGAGGCGGAGGAGAACAA
	Reverse	AGGCGGTAGTAGGACAGGA
Human p21	Forward	GGCAGACCAGCATGACAGATT
	Reverse	GCGGATTAGGGCTTCCTCTT
Human GAS	Forward	GCTAGCCGCTACCTGACC
	Reverse	CCTTGGGCATAGCCAGCAT
Human B2M	Forward	TCACCCCCACTGAAAAAGATGAGTA
	Reverse	GAATTCTCTGCTCCCCACCTCTAAG
Human EEF1A1	Forward	TGTCGTCATTGGACACGTAGA
	Reverse	ACGCTCAGCTTTCAGTTTATCC
Human RPS6	Forward	TCTTGACCCATGGCCGTGTC
	Reverse	GCGGCGAGGCACTGTAGTAT
Human ACTIN	Forward	GATGAGATTGGCATGGCTTT
	Reverse	CACCTTCACCGTTCCAGTTT
